# Variability in mRNA expression of *fms-like tyrosine kinase-1* variants in normal and preeclamptic placenta

**DOI:** 10.1186/1756-0500-7-154

**Published:** 2014-03-17

**Authors:** Laura Surmon, Gabriele Bobek, Angela Makris, Christine L Chiu, Craig A Lind, Joanne M Lind, Annemarie Hennessy

**Affiliations:** 1 School of Medicine, University of Western Sydney, Building 30, Campbelltown Campus, Locked Bag 1797, Penrith, NSW 2751, Australia; 2 The Heart Research Institute, University of Sydney, Sydney, NSW, Australia; 3 Liverpool Hospital, Liverpool, NSW, Australia

**Keywords:** Placenta, Preeclampsia, sFLT-1, Variability, Sampling

## Abstract

**Background:**

Preeclampsia is a complication of pregnancy characterised by gestational hypertension and proteinuria and is a leading cause of morbidity and mortality in both mothers and infants. Certain anti-angiogenic factors have long been implicated in the pathogenesis of preeclampsia and the placental expression of factors such as soluble fms-like tyrosine kinase-1 (sFLT-1) are often reported in studies of normal and diseased placentae. Despite evidence showing significant differences in placental gene expression by collection site, many studies fail to provide sufficient details on sample selection and collection.

**Findings:**

With ourselves and others investigating and reporting on the expression of *FLT-1* variants and other genes in the placenta of normotensive and preeclamptic patients, we felt it prudent to examine the variation in expression of *FLT-1* variants across human placenta. We examined the differential expression of *FLT-1* variants in samples obtained from 12 sites on normal and preeclamptic placentae and found expression to be highly variable between sites. We therefore developed an algorithim to calculate the mean expression for any number of these sites collected and in any combination. The coefficient of variation for all combinations of sites was then used to determine the minimum number of sites required to reduce coefficient of variation to below an acceptable 10%. We found that 10 and 11 sites had to be sampled in the normal and preeclamptic placentae respectively to ensure a representative expression pattern for all *FLT-1* variants for an individual placenta.

**Conclusions:**

These findings demonstrate significant variation in expression levels of several commonly investigated genes across sites in both normal and preeclamptic placenta. This highlights both the importance of adequate sampling of human placenta for expression studies and the effective communication of sample selection and collection methods, for data interpretation and to ensure the reproducibility and reliability of results and conclusions drawn.

## Findings

### Background

Preeclampsia is a complication of pregnancy characterised by gestational hypertension and proteinuria and is a leading cause of morbidity and mortality in both mothers and infants [1]. Reduced trophoblast invasion and spiral artery remodelling in early pregnancy leads to the abnormal placental development and reduced placental perfusion seen in preeclampsia [1]. The clinical manifestations of this syndrome become evident when the abnormal placenta releases different placental factors into the maternal circulation [2]. Soluble fms-like tyrosine kinase-1 i13 (sFLT-1 i13) and its splice variant sFLT-1 e15a/sFLT-1 14 have been shown to be increased in the serum and placental tissue of women with preeclampsia [1-5]. sFLT-1 is the soluble form of the vascular endothelial growth factor (VEGF) receptor-1 [6,7] that binds VEGF and other angiogenic factors [8], preventing their interaction with the membrane-bound form of FLT-1 (mFLT-1) [4]. This imbalance of pro- and anti-angiogenic factors in the maternal circulation leads to the widespread endothelial dysfunction characteristic of preeclampsia [9]. Studies reporting on human placental gene expression often fail to provide detailed information on how tissue samples were selected and collected [10], despite evidence showing significant differences in placental gene expression depending on collection site [10]. No studies have measured the variation in expression of *FLT-1* variants across human placenta. This study aimed to measure the expression of *FLT-1* variants from different collection sites within normal and preeclamptic placentae and determine the minimum number of sites per placenta that should be sampled to ensure reproducibility and reliability of results.

### Methods

#### Placental tissue collection

Placentae were collected from normotensive (n = 3) and preeclamptic (n = 2) women following caesarean section of female babies at Campbelltown Public Hospital, NSW, Australia (Table 1). Both preeclamptic women were diagnosed prior to 34 weeks gestation. Samples were obtained from each placenta within 30 minutes of delivery on ice. Random sampling was used to excise 12 circular segments (15 mm diameter × 2 mm depth) from sites around the decidual surface at least 30 mm apart. Regions of calcification were avoided and no decidual contamination was found. This study was approved by the Sydney Local Health District Ethics Review Committee and informed, written consent was obtained from each patient.

**Table 1:** Patient Characteristics

**Placenta**	**Pregnancy outcome**	**Medication**	**Age (years)**	**Gestational age at delivery (weeks)**	**Birth weight (g)**
NP1	Normotensive	Nil	39	39.1	3255
NP2	Normotensive	Nil	33	38.6	4470
NP3	Normotensive	Nil	31	39.4	4315
PE1	Preeclamptic	Labetolol and Aldomet	30	36.9	3840
PE2	Preeclamptic	Labetolol and Aldomet	24	37.4	3210

#### Tissue homogenisation, RNA extraction and cDNA synthesis

Individual tissue samples were homogenised in the deep frozen state and total RNA extracted using the RNAeasy Plus Mini Kit [Qiagen, Hilden, Germany] as per the manufacturer’s protocol. cDNA was synthesised from 1 μg RNA using the Affinity Script qPCR cDNA Synthesis kit [Stratagene, La Jolla, CA, USA] according to the manufacturer’s protocol.

#### Quantitative PCR (qPCR)

Quantitative PCR (qPCR) and published primer sequences [5] were used to measure mRNA expression of *mFLT-1*, *sFLT-1 i13* and *sFLT-1 e15a* in placental tissue. Beta actin (*bAct*) was used as a normaliser gene (forward primer 5′- TTCGGAAGACAGAAGTTCTCGTT -3′ and reverse primer 5′- GACCTCGTAGTCACTGAGGTTTTG -3′). Individual reactions (10 μl) contained GoTaq® Flexi Buffer, 2.5 mM MgCl_2_ and 1.25 U GoTaq® DNA Polymerase [Promega, Madison, WI, USA], 0.4 mM dNTPs [Bioline, Boston, MA, USA], Sybr® Green I [Sigma Aldrich, Mannheim, Germany], dimethylsulphoxide [Sigma Aldrich], forward and reverse primers (0.5 μM), and 4 μl of cDNA (4 μl water for no-template controls). A pooled sample of cDNA was used to generate a standard curve for each primer set, and the efficiency of the reaction was calculated. Reactions were carried out in a MxPro3005P Real Time PCR System [Stratagene] under the following conditions: 95°C 10 min; 95°C 30 sec, 60°C 30 sec, 72°C 30 sec for 40 cycles; followed by a dissociation curve. All samples were run in triplicate.

#### Statistical analyses

Triplicate Ct values were averaged and the fold change of each sample calculated using the delta-delta Ct method, normalised to *bAct* expression [11]. Fold changes were log transformed prior to statistical analysis.

Within a placenta, the first sample collected served as the reference sample and expression for the remaining 11 samples was calculated relative to this sample. The resulting fold-change data was used for subsequent steps. To determine the minimum number of sites on a placenta that must be sampled to achieve a coefficient of variation (CV) below 10% for each transcript, all possible combinations of sites had to be considered. To achieve this, we developed a web-based algorithm that took every possible set of 2 samples (66 in total) and calculated the mean of each (http://iconvm.com.au/jonomics). The %CV was then calculated for the 66 means generated. This was repeated using every possible set of 3 samples (220 combinations in total) and so on up to 12 samples, each time calculating the %CV for the set of means generated. The %CV was then plotted against the number of sites to determine the minimum number of sites required to obtain a %CV of less than 10%.

### Results

**Figure 1:**
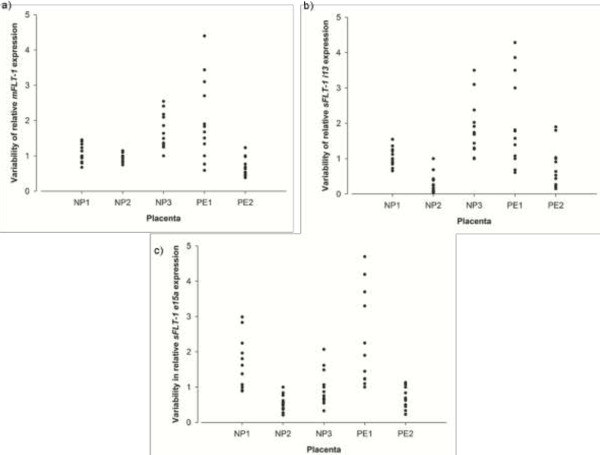
**Variability in relative transcript expression across different sites on normal and preeclamptic placentae. (a) ***mFLT-1*, **(b)***sFLT-1 i13* and **(c)***sFLT-1 e15a.*

The expression of *mFLT-1, sFLT-1 i13*, and *sFLT-1 e15a* across the 12 collection sites within a placenta was highly variable, with a greater than six-fold difference in expression between sites on the same placenta (Figure 1). Transcript expression was consistently more variable within the preeclamptic placentae compared to the normal placentae (Figure 1).

**Figure 2:**
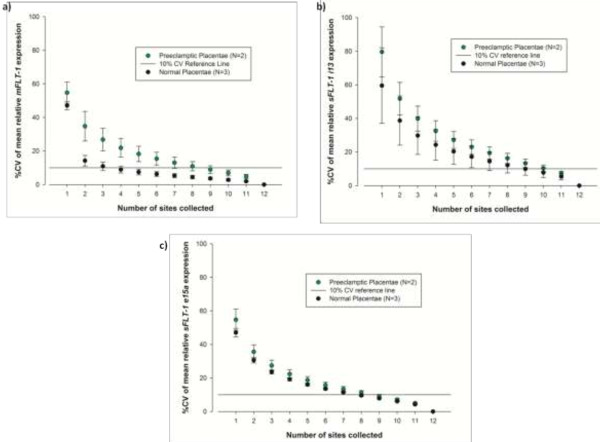
**Percentage coefficient of variation by number of sites collected on normal and preeclamptic placentae.** To determine the minimum number of sites on a placenta that must be sampled to achieve a coefficient of variation (CV) below 10% for each transcript, all possible combinations of sites had to be considered. To achieve this, we developed a web-based algorithm that took every possible set of 2 samples (66 in total) and calculated the mean of each (http://iconvm.com.au/jonomics). The %CV was then calculated for the 66 means generated. This was repeated using every possible set of 3 samples (220 combinations in total) and so on up to 12 samples, each time calculating the %CV for the set of means generated. This figure is a plot of %CV against the number of sites. The minimum number of sites required to obtain a %CV of less than 10% for both normal and preeclamptic placenta is shown for **(a)***mFLT-1*, **(b)***sFLT-1 i13* and **(c)***sFLT-1 e15a.*

A minimum of 10 and 11 sites per normal and preeclamptic placentae respectively needed to be sampled in order to achieve a CV below 10% for all transcripts (Figure 2).

### Discussion

This study demonstrates that the expression of *mFLT-1*, *sFLT-1 i13* and *sFLT-1 e15a* can vary significantly within a placenta. We show that of the placentas included in our study, a minimum of 10 sites had to be sampled in the normal placenta and 11 sites in the preeclamptic placenta in order to ensure a representative expression pattern for an individual placenta.

The expression of *mFLT-1* was less variable in normal placenta and likely reflects the more stable nature of the expression of this membrane-bound variant under different conditions [12]. This is the first report of more stable mFLT-1 expression in normotensive placentas, compared with placentas from women with preeclampsia. This is in contrast to the soluble, anti-angiogenic factors sFLT-1 i13 and sFLT-1 e15a, which have long been implicated in the pathogenesis of preeclampsia [1-6,13], with the expression of these genes shown to differ between healthy and preeclamptic placentae. A diagnosis of preeclampsia for research purposes often relies on the differential expression of these genes comparing normal and preeclamptic placentae, however these studies do not report how many sites have been sampled within a placenta [10]. We have shown there is significant variation in gene expression within an individual placenta, indicating that under-sampling can result in misleading data. We therefore recommend that samples be obtained from no less than 11 separate sites when pooling placental samples for expression analysis and that sampling strategy details be reported in the methods. We would also recommend that these methods be followed for other genes of interest in human placenta to determine the minimum number of sites required for a representative expression pattern, and we have made our algorithm publicly available for this purpose.

## Availability of algorithm

Web address: http://iconvm.com.au/jonomics

Access: Freely available for use

Instructions:

1. Copy and paste data from Excel/SPSS etc. into number generator.

2. Click ‘Process Results’.

3. A results summary will show the number of all possible combinations for each possible set size for your data.

4. Select ‘Download Results (CSV)’ to import the results to Microsoft Excel.

## Competing interests

The authors declare that they have no competing interests.

## Authors’ contributions

LS conceived the study, participated in its design and coordination, performed all laboratory work and participated in all statistical analysis. GB participated in design and coordination of the study and helped draft the manuscript. AM participated in design of the study and edited the manuscript. CC participated in study design, statistical analysis and helped draft the manuscript. CL developed the web-based algorithm used for data analysis. JL participated in data interpretation, statistical analysis and helped draft the manuscript. AH participated in study design and coordination. All authors read, edited where necessary and approved the final manuscript.
